# Development of a duplex TaqMan real-time RT-PCR assay for simultaneous detection of newly emerged H5N6 influenza viruses

**DOI:** 10.1186/s12985-019-1229-2

**Published:** 2019-10-22

**Authors:** Lin Liu, Ying Zhang, Pengfei Cui, Congcong Wang, Xianying Zeng, Guohua Deng, Xiurong Wang

**Affiliations:** National Avian Influenza Reference Laboratory, State Key Laboratory of Veterinary Biotechnology, Harbin Veterinary Research Institute, Chinese Academy of Agricultural Sciences, Harbin, 150069 China

**Keywords:** Duplex rRT-PCR, H5N6 virus, Avian influenza virus

## Abstract

**Background:**

In 2017–2018, a new highly pathogenic H5N6 avian influenza virus (AIV) variant appeared in poultry and wild birds in Asian and European countries and caused multiple outbreaks. These variant strains are different from the H5N6 virus associated with human infection in previous years, and their genetic taxonomic status and antigenicity have changed. Therefore, revision of the primers and probes of fluorescent RT-PCR is important to detect the new H5N6 subtype AIV in poultry and reduce the risk of an epidemic in birds or humans.

**Methods:**

In this study, the primers and probes including three groups of HA and four groups of NA for H5N6 influenza virus were evaluated. Then a set of ideal primer and probes were selected to further optimize the reaction system and established a method of double rRT-PCR assay. The specificity of this method was determined by using H1~H16 subtype AIV.

**Results:**

The results showed that fluorescence signals were obtained for H5 virus in FAM channel and N6 virus in VIC channel, and no fluorescent signal was observed in other subtypes of avian influenza viruses. The detection limit of this assay was 69 copies for H5 and 83 copies for N6 gene. And, the variability tests of intra- and inter-assay showed excellent reproducibility. Moreover, this assay showed 100% agreement with virus isolation method in detecting samples from poultry.

**Conclusion:**

The duplex rRT-PCR assay presented here has high specificity, sensitivity and reproducibility, and can be used for laboratory surveillance and rapid diagnosis of newly emerged H5N6 subtype avian influenza viruses.

## Background

Avian influenza viruses (AIVs) belong to the Orthomyxoviridae family with a natural reservoir almost entirely in birds [1]. The virus particle contains a genome that includes eight separate negative-stranded RNA segments and majorly adopts an elliptical shape [2]. The two large proteins found on the surface of the viral envelope are hemagglutinin (HA) and neuraminidase (NA) [3]. Based on the antigenicity of these two proteins, AIVs are categorized into different subtypes [4]. Currently, 16 different HA and 9 different NA subtypes of AIVs have been identified [5].

H5 subtype AIVs can be classified into two groups based on their pathogenicity on chickens: high pathogenicity and low pathogenicity groups [6]. Highly pathogenic H5 subtype AIVs have infected wild birds, and continued to cause outbreaks in poultry [7]. Besides, these viruses have also caused sporadic infections in humans, thus posing serious threats to public health [8]. Influenza viruses have a relatively high mutation rate due to the low fidelity of RNA polymerase [9–11]. And the segmented nature of its genome promotes the reassortment of viral genes in hosts that were simultaneously infected with two different influenza viruses [12–14]. In recent years, these H5 viruses have diversified genetically and antigenically, including the emergence of viruses with replacement of the N1 gene segment by N2, N3, N5, N6, N8 or N9 gene segments [7, 15]. The highly pathogenic H5N6 influenza viruses emerged in poultry in Asia, especially Southeast Asia, in 2013 [16–19]. Since then these viruses have gradually replaced the H5N1 virus to become the dominant strains.

In 2017–2018, a new highly pathogenic H5N6 AIV variant appeared in poultry and wild birds in Asian and European countries and led to multiple outbreaks [20]. These variant strains are different from the H5N6 virus associated with human infection in previous years, and their genetic taxonomic status and antigenicity have all changed [21]. A new strain of this H5N6 virus has been detected in migratory birds and is likely to pose a potential threat to domestic poultry in Asia, Europe as well as the world, through wild bird transmission. Therefore, it is very important to revise the real time RT-PCR detection method for H5N6 subtype AIV to prevent and control the spread of H5N6 AIV. In this study, a fast, specific, and sensitive TaqMan real time RT-PCR method was established for the diagnosis of the newly emerged H5N6 viruses.

## Method

### Reagents

One-step real time RT-PCR master mixes were obtained from Thermo Fisher Scientific (Waltham, MA). TIANamp virus RNA kit was purchased from Tiangen Biotech (Beijing, China).

### Viral RNA extraction

The extraction of viral RNA of the H5N6 viruses was performed according to the manufacturer’s instructions in the BSL3 laboratory at Harbin Veterinary Research Institute (HVRI), Chinese Academy of Agricultural Sciences. Viral RNA concentration was measured with a NanoDrop ND-2000 apparatus (Thermo Scientific, Wilmington, DE).

### Primer and probe design

Primers and Probes were designed based on the highly conservative regions of HA and NA genes of A/Duck/Guangxi/1/18(H5N6) virus downloaded from GenBank by using the MegAlign module of the DNAStar package. One H5 HA primer (H5A), four NA primers and TaqMan probes pertaining to them were designed by use of the Primer Express 3.0 software, and were screened by using the blast function of NCBI to ensure their specificity. Two H5 HA primers and probes were cited from reference (H5B and H5C) [22]. The probes pertaining to HA labelled with FAM and BHQ1 quencher group in the 5′ and 3′ terminus, and the probes pertaining to NA labelled with VIC and BHQ1 quencher group in the 5′ and 3′ terminus (Table 1) were synthesized by Sangon Biotech (Shanghai) Co., Ltd.

**Table 1:** Primers and probes used for the assay

Name	Sequences(5′-3′)
H5A-F	AGG GAG GAT GGC AGG GAA TG
H5A-R	TCT TTG TCT GCA GCG TAC CCA CT
H5A-P	FAM- ATG GTT GGT ATG GGT ACC ACC ATA GCA ATG -BHQ1
H5B-F	TCC TTG CAA CAG GAC TAA G
H5B-R	GTC TAC CAT TCC ATG CCA
H5B-P	FAM- AAG AAR AAA RAG AGG ACT RTT TGG AGC T-BHQ1
H5C-F	ATA CCA GGG AAC GCC CTC M
H5C-R	ATT ATT GTA GCT TAT CTT TAT TGT Y
H5C-P	FAM- TCG TTC TTT TTG ATG AGC CAT ACC ACA-BHQ1
N6A-F	TGC AGG ATG TTT GCT CTG AGT C
N6A-R	CGA AAT GGG CTC CTA TCA TGT AT
N6A-P	VIC- ACA ACA CTC AGA GGG CAA CAT GCG AAT -BHQ1
N6B-F	ACC GAC ACA AGT CGT CCT AAT GA
N6B-R	ACC CTT TTA CCC CTG GGT CTG
N6B-P	VIC - ATG GGA ACT GCG ATG CGC CAA TAA C-BHQ1
N6C-F	AAG GTC CCA AAT GCA GAA ACC
N6C-R	CCC TGA GTA CCC TGA CCA ATT TT
N6C-P	VIC -ACA CTC AAT CAG GGC CAA CCT CAT ACC A-BHQ1
N6D-F	CAG GGG TAA AAG GGT TTG CAT
N6D-R	TGC ATT TGG GAC CTT TAA CAT TTC
N6D-P	VIC - ACA CTC AAT CAG GGC CAA CCT CAT ACC A-BHQ1
H5-200F	AAT ACC CCT CAG AGA GAG AGA AG
H5-200R	ATC GAG TTG ACC TTA TTG GTG
N6-267F	GGC ACA TTC TAT CGA AAG ACA ATG C
N6-267R	CAT GGC ATG ACG TGC TTG AC A

### Reference strain

#### Viruses

H1-H16 reference influenza viruses and the highly pathogenic H5N6 virus strain, A/Duck/Guangxi/1/18(H5N6), were stored in the National Avian Influenza Reference Laboratory at HVRI. The Newcastle disease virus (NDV) strains (F48E9 and LaSota), avian infectious bronchitis virus (IBV), and infectious bursal disease virus (IBDV) (Table 2), were obtained from the corresponding research teams of State Key Laboratory of Veterinary Biotechnology at HVRI.

**Table 2:** Reference strains used in this experiment and specificity results of the TaqMan real time RT-PCR

Name of reference strains	Subtype	Ct/H5-FAM	Ct/N6-VIC
A/mallard/Sanjiang/390/2007	H1N1	undet	undet
A/mallard/Heilongjiang/135/2006	H2N2	undet	undet
A/mallard/Heilongjiang/90/2006	H3N2	undet	undet
A/duck/Guangxi/S-2-248/2009	H4N6	undet	22.05
A/Duck/GuangXi/1/2018	H5N6	15.87	14.97
A/mallard/Heilongjiang/81/2006	H6N2	undet	undet
A/equine/ jingfang/74–1	H7N7	undet	undet
A/turkey/ontario/6118/1968	H8N4	undet	undet
A/Turkey/Wisconsin/1/66	H9N2	undet	undet
A/Turkey/England/384/1979	H10N4	undet	undet
A/Duck/Memphis/546/1976	H11N9	undet	undet
A/duck/Alberta/60/1976	H12N5	undet	undet
A/gull/Maryland/704/1977	H13N6	undet	17.9
A/mallard/Gurjer/263/1982	H14N5	undet	undet
A/duck/Australia/341/83	H15N8	undet	undet
A/cormorant/Denmark/74–68,899-G2/02	H16N3	undet	undet
NDV	LaSota	undet	undet
NDV	F48E9	undet	undet
IBV	Lx4	undet	undet
IBDV	Gt	undet	undet

#### TaqMan real time RT-PCR

One-step real time RT-PCR master mix was used to perform the real time RT-PCR in a 25 μL reaction volume, including 12.5 μL 2 × RT-PCR buffer, 1.25 μL enhancer, 4.05 μL RNase free ddH_2_O, 0.25 μL RT-PCR enzyme mixture, 3.5 μM HA forward primer, 3.5 μM HA reverse primer, 1.5 μM HA probe, 8 μM NA forward primer, 8 μM NA reverse primer, 5 μM NA probe, and 4 μL sample RNA. The reaction was conducted with the following conditions: an initial cycle at 50 °C for 15 min, one cycle at 95 °C for 15 min, followed by 40 cycles of 95 °C for 15 s, 60 °C 45 s. The FAM and VIC fluorescence signals were collected after each annealing step. Based on the real-time PCR amplification curve, a proper set of primers and probes were selected.

#### Specificity of TaqMan real time RT-PCR

Viral RNA was extracted from all viruses listed in Table 2 with the TIANamp virus RNA kit, and ddH_2_O was used as a negative control in the experiment. All samples were tested under the PCR reaction system and conditions described above for verifying the specificity of the established TaqMan real time RT-PCR method.

#### Sensitivity of TaqMan real time RT-PCR

The viral RNA of A/Duck/Guangxi/1/18(H5N6) virus was subject to RT-PCR with the primer pairs H5-200F/H5-200R and N6-267F/ N6-267R. The HA and NA PCR products were inserted into the pMD 18-T vector and sequenced. The recombinant plasmids were measured for its concentration and then subject to 10-fold serial dilution with ddH_2_O. The dilutions from 10^− 1^ to 10^− 10^ were used to test the sensitivity of the rRT-PCR method established above.

#### Comparison with the method of virus isolation from embryonated eggs

15 six-week-old SPF chickens were randomly divided into three groups of 5 each. Group 1 and 2 were inoculated intranasally with a 0.2 mL total dose containing 3 and 30 EID_50_ of H5N6 highly pathogenic challenge virus, respectively. The 5 non-infected chickens were inoculated with 0.2 mL PBS as negative controls. Oropharyngeal and cloacal swabs were collected from all chickens at 3 days post infection for comparison with the method of virus isolation using embryonated chicken eggs. Independently, fifty-two clinical samples, including heart, liver, spleen, lung, kidney, trachea, intestines, oropharyngeal and cloacal swabs were collected from chickens of suspicious clinical infection, and were examined by using rRT-PCR and virus isolation method, separately.

## Results

### Selection of optimal pair of primers and probe

**Fig. 1:**
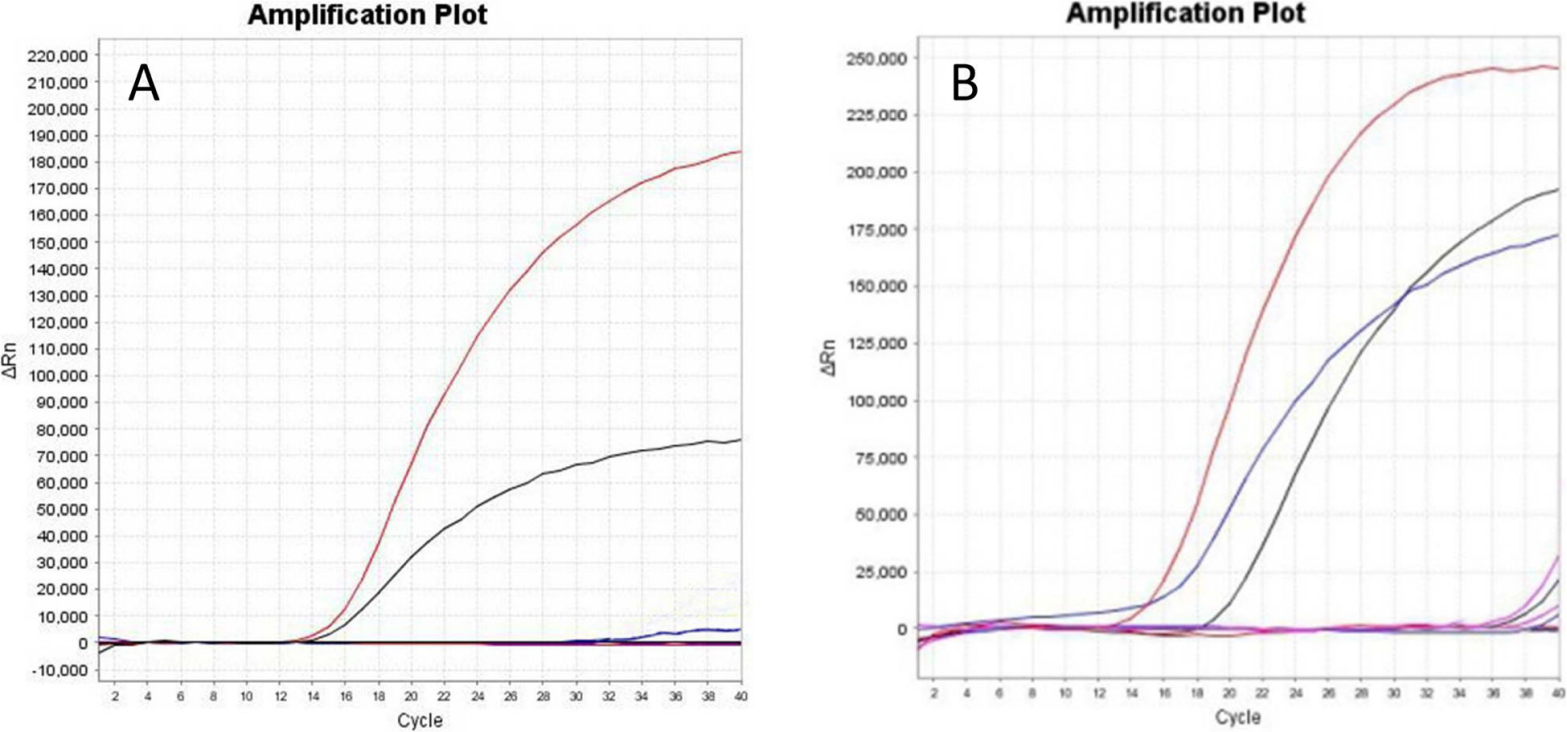
**a** The amplification results of H5A-F/H5A-R/H5A-P, H5B-F/H5B-R/H5B-P, and H5C-F/H5C-R/H5C-P primers and probes. The amplification curves for primer/probe sets H5A-F/H5A-R/H5A-P, H5B-F/H5B-R/H5B-P, and H5C-F/H5C-R/H5C-P are shown in red line, black line and blue line, respectively. **b** The amplification results of N6A-F/N6A-R/N6A-P, N6B-F/N6B-R/N6B-P, N6C-F/N6C-R/N6C-P and N6D-F/N6D-R/N6D-P primers and probes. The amplification curves for primer/probe sets N6A-F/N6A-R/N6A-P, N6B-F/N6B-R/N6B-P, N6C-F/N6C-R/N6C-P and N6D-F/N6D-R/N6D-P are shown in red line, blue line, black line, purple line, respectively

Three different pairs of primers and four probes (Table 1) were used to perform TaqMan real time RT-PCR with the viral RNA of A/Duck/Guangxi/1/18(H5N6) as template. The amplification with H5A-F/H5A-R/H5A-P and N6A-F/N6A-R/N6A-P showed the highest fluorescent signal increment, while no amplification peak was observed in the negative ddH_2_O control. Therefore, set of H5A-F/H5A-R/H5A-P (Fig. 1a) and N6A-F/N6A-R/N6A-P (Fig. 1b) primers and probe were selected for the follow-up experiment.

### The established TaqMan real time RT-PCR method exhibited high specificity

The specificity of rRT-PCR assay was analyzed by amplification of viral RNAs extracted from viruses in Table 2 with primers and probes selected above. We found that only the amplification with A/Duck/Guangxi/1/18(H5N6) viral RNA produced two standard amplification curves, H4N6 and H13N6 viral RNA showed one standard amplification curve, whereas amplification of all other samples resulted in a sign of lack of amplification (Table 2). This result indicates that the established rRT-PCR method is highly specific for detecting the viral RNA of highly pathogenic H5N6 virus.

### The established TaqMan real time RT-PCR method are highly sensitive

**Fig. 2:**
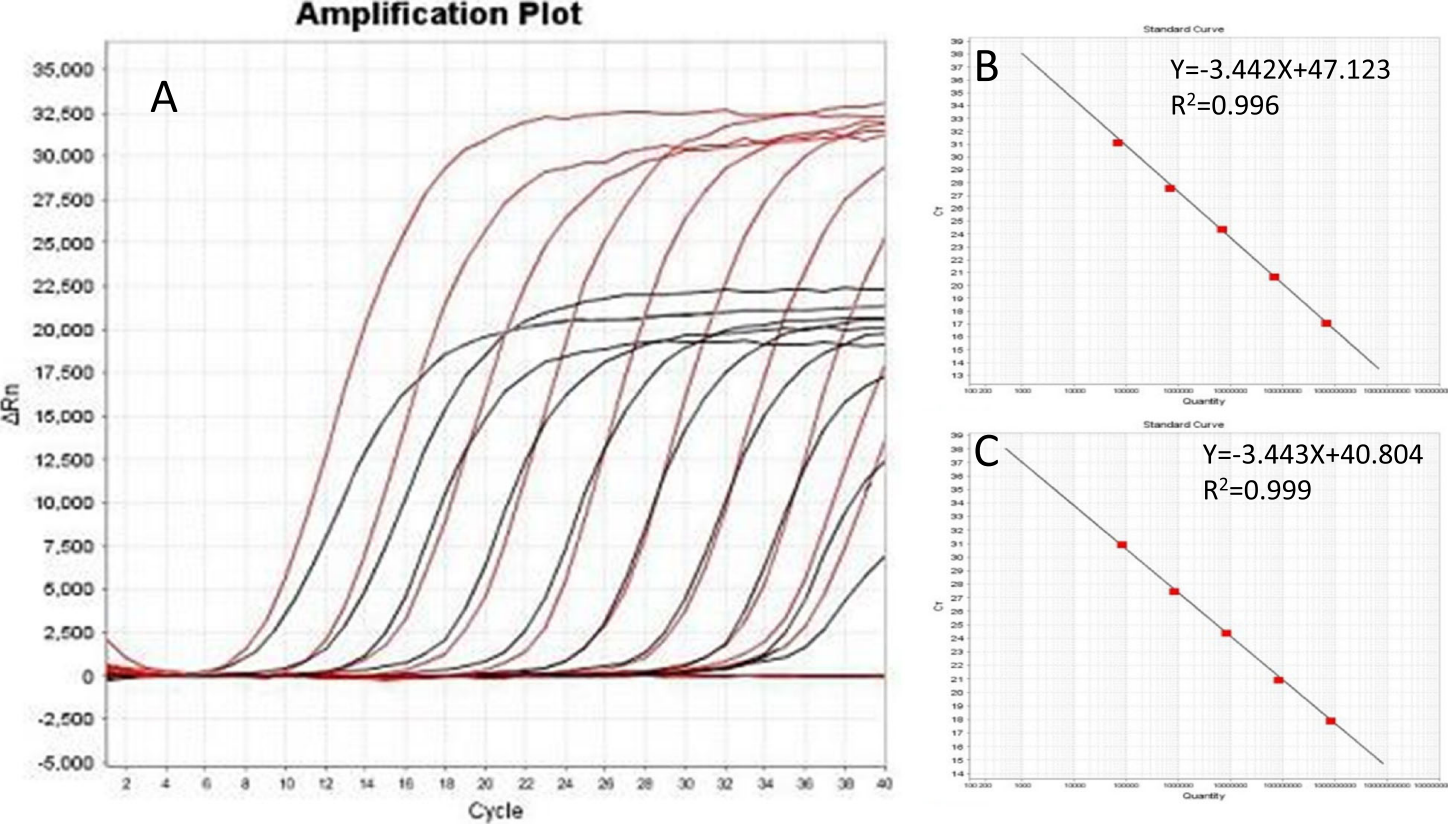
The hemagglutinin (HA) and Neuraminidase (NA) plasmids of a highly pathogenic H5N6 virus (DK/GX/1/18) were 10-fold serially diluted with ddH_2_O, and 10^−1^–10^−10^ dilutions were used to evaluate the TaqMan real-time RT-PCR method. Amplification curves are shown in the left panel in descending order of the template concentration with the highest concentration (10^− 1^) on the left. **a**. The standard curve is shown in the right panel. The pHA plasmid displayed a slope of −3.442, an intercept of 47.123, a correlation coefficient of 0.996, and an amplification efficiency of 95.218% **b**. The pNA plasmid dilutions from 10^− 3^ to 10^−7^ displayed a slope of − 3.443, an intercept of 40.804, a correlation coefficient of 0.999, and an amplification efficiency of 95.192% (**c**)

A 200 bp HA fragment and 267 bp NA fragment of the A/Duck/Guangxi/1/18(H5N6) virus were amplified with the primer pairs H5-200F/H5-200R and N6-267F/ N6-267R, then were cloned into the pMD 18-T vector. The two constructed plasmids were confirmed by sequencing and named as pHA and pNA, respectively. The concentration of the pHA plasmid was 219.1 μg/μL and was calculated to contain 6.9 × 10^10^ copies /mL. The concentration of the pNA plasmid was 270.3 μg/μL and was calculated to contain 8.3 × 10^10^ copies /μL. Two plasmids were ten-fold serially diluted with ddH_2_O, and the dilutions from 10^− 1^~10^− 10^ were subjected to rRT-PCR amplification. We found that the fluorescent signal can be detected in 10^− 10^ dilutions with as low as 69 copies per reaction for the H5 and 83 copies for the N6 genes (Fig. 2a). In addition, the amplification curves of pHA plasmid dilutions from 10^− 3^ to 10^− 7^ are well linearly correlated, displaying a slope of − 3.442, an intercept of 47.123, a correlation coefficient of 0.996, and an amplification efficiency of 95.218% (Fig. 2b). The amplification curves of pNA plasmid dilutions from 10^− 3^ to 10^− 7^ are also well linearly correlated, displaying a slope of − 3.443, an intercept of 40.804, a correlation coefficient of 0.999, and an amplification efficiency of 95.192% (Fig. 2c). The 10^− 9^ and 10^− 10^ dilutions showed similar Ct values, so we set up the cut-off value as 35.

### Reproducibility of the real-time PCR

Standard samples in 3 different concentrations (3 × 10^9^copies/μl, 3 × 10^7^copies/μl, and 3 × 10^5^copies/μL) were used in intra- and inter-assay comparisons for testing the reproducibility of the rRT-PCR method. The variations in the inter-assay were evaluated by testing 4 replicates of each concentration in a single round of real-time PCR, while 4 independent experiments were performed to test the variations of the intra assay. The coefficients of variation (CV) for the Ct values of the intra- and inter-assay comparisons were listed in Table 3.

**Table 3:** Intra- and Inter-assay coefficients of variation for duplex TaqMan Real-time RT-PCR

Dilution of RNA	n	Intra-assay(%)		Inter-assay(%)	
		H5	N6	H5	N6
10^−1^	4	1.12%	1.83%	1.41%	1.68%
10^−3^	4	1.50%	1.71%	1.25%	2.29%
10^−5^	4	1.42%	1.31%	1.11%	1.13%

### Detecting samples from experimentally or naturally infected chickens

Twenty swab samples were collected from the SPF chickens inoculated intranasally with H5N6 virus. All of the twenty samples were identified to be positive by the rRT-PCR method (Table 4). In contrast, the swab samples of the ten non-infected chickens were test-negative through both rRT-PCR and virus isolation method. Besides, fifty-two field samples collected from chickens showing influenza-like symptoms were selected to further test the efficacy of the established rRT-PCR method. Among them, four samples were positive for the highly pathogenic H5N6 virus, while others were negative, which showed 100% of consistency with the virus isolation method using embryonated chicken eggs.

**Table 4:** Virus detection from oropharyngeal and cloacal swabs of the experimentally infected chickens by real-time PCR and virus isolation

	Control		Infected group 1 with 3 EID50		Infected group 2 with 30 EID50	
	H5	N6	H5	N6	H5	N6
Oropharyngeal Swabs	undet	undet	27.18	23.15	25.41	22.27
	undet	undet	25.16	22.33	27.63	24.1
	undet	undet	22.89	19.59	23.28	20.33
	undet	undet	32.32	29.39	27.02	22.2
	undet	undet	31.25	32.11	28.63	25.08
Cloacal Swabs	undet	undet	28.52	21.93	23.71	20.28
	undet	undet	22.83	19.13	27.05	23.86
	undet	undet	25.86	21.87	27.65	24.03
	undet	undet	32.21	33.95	25.9	21.34
	undet	undet	31.43	27.94	24.51	20.8

## Discussion

According to a report from the World Organization for Animal Health (OIE), the initially described outbreaks in poultry caused by an H5N6 HPAIV were in China and Lao People’s Democratic Republic in early May 2014 [23]. Since then, H5N6 HPAIV has emerged in Vietnam, Hong Kong, Japan, South Korea and other countries [24–26]. H5N6 HPAIV causes severe clinical symptoms and extremely high mortality in poultry, and a large number of poultry are exterminated in order to control the epidemic. The H5N6 HPAIV is also detected in some human patients with severe pneumonia and even causes death. A total of 19 laboratory-confirmed cases of human infection with H5N6 virus, including six deaths, have been reported to WHO from China since 2014 (http://www.wpro.who.int/emerging_diseases/ai_weekly_626_wpro_20180302.pdf). Thus, H5N6 HPAIV not only threatens the healthy development of the poultry industry, but also poses significant public health risks.

The development of rapid and accurate influenza virus detection methods is of great significance for preventing influenza virus outbreaks. To date, a series of diagnostic methods have been established for the diagnosis of influenza virus, such as virus isolation, RT-PCR, real-time RT-PCR, and rapid influenza diagnostic tests (RIDTs). Among them, real-time RT-PCR, which generated the target-specific fluorescence signal based on the hydrolysis probe or“TaqMan” method, has become one of the most commonly used method because of its high sensitivity. Currently, TaqMan real-time RT-PCR protocols have been developed for the detection and typing of H5, H7 and H9 RNA [27].

In this experiment, a one-step duplex real-time RT-PCR assay was developed to identify H5N6 subtype AIVs. The advantages of a duplexed assay are as follows: low cost, low risk of carryover contamination, and reduced hands-on time. Contrasting to single probe rRT-PCR, this method introduced two different labeled probes so that it can simultaneously detect two kinds of targets to improve processing efficiency. The specific probes labeled with different fluorescent dyes were used to distinguish the H5N6 AIVs. Only H5N6 subtype virus has double fluorescent signals resulting from this assay, H5 or N6 AIVs from other lineages can also be detected by this assay, and other avian respiratory viruses and negative allantoic fluid have no signal. If only H5 genes were detected while N6 genes were not, it indicated that other H5 AIV lineages were in the samples. Likewise, if only N6 genes were detected while H5 genes were not, it meant that other N6 subtype AIVs were in the samples. The limit of the detection was as low as 69 copies per reaction for the H5 and 83 copies for the N6 genes respectively. In the meantime, excellent results were obtained in the reproducibility test.

In addition, three set of primers and probes for H5 HA were analyzed in this experiment. The results showed that two sets of primers and probes, used for the detection of early period H5 AIVs, showed no efficient amplification when testing the HA of newly emerged H5N6 virus. It meant that due to the diversity of viruses from different avian species, distinct geographic areas, and variable time periods, each set of primers and probes should be further verified.

## Conclusions

The duplex TaqMan real-time RT-PCR assay developed in the present study offers a useful option for laboratories requiring a high-throughput testing capacity. It allows for the simultaneous detection of H5 HA and N6 NA within influenza virus samples, which is both cost and time effective. Its testing efficiency is improved by decreasing the volume of pipetted sample required, reducing the cost of reagents, and increasing the number of samples tested in the same run. This may be particularly useful in the post-pandemic period with heightened awareness and continued surveillance.

## Data Availability

Please contact author for data requests.

## Abbreviations

AIV: Avian Influenza Virus
BSL3: Biosafety Laboratory 3
EID50: 50% Egg Infectious Dose
HPAIV: Highly Pathogenic Avian Influenza Virus
HVRI: Harbin Veterinary Research Institute
IBDV: Infectious Bursal Disease Virus
IBV: Avian Infectious Bronchitis Virus
NDV: Newcastle Disease Virus
OIE: World Organization for Animal Health
PBS: Phosphate Buffer Saline
RIDTs: Rapid Influenza Diagnostic Tests
rRT-PCR: Real time Reverse Transcription-Polymerase Chain Reaction
SPF: Specific Pathogen Free
WHO: World Health Organization

## References

[1] Liu L, Zeng X, Chen P, Deng G, Li Y, Shi J (2016). Characterization of clade 7.2 H5 avian influenza viruses that continue to circulate in chickens in China. J Virol.

[2] Li C, Chen H (2014). Enhancement of influenza virus transmission by gene reassortment. Curr Top Microbiol Immunol.

[3] Webster RG, Govorkova EA (2014). Continuing challenges in influenza. Ann N Y Acad Sci.

[4] Li C, Bu Z, Chen H (2014). Avian influenza vaccines against H5N1 'bird flu'. Trends Biotechnol.

[5] Hiono T, Okamatsu M, Matsuno K, Haga A, Iwata R, Nguyen LT (2017). Characterization of H5N6 highly pathogenic avian influenza viruses isolated from wild and captive birds in the winter season of 2016-2017 in Northern Japan. Microbiol Immunol.

[6] Li M, Zhao N, Luo J, Li Y, Chen L, Ma J (2017). Genetic characterization of continually evolving highly pathogenic H5N6 influenza viruses in China, 2012-2016. Front Microbiol.

[7] Bi Y, Chen Q, Wang Q, Chen J, Jin T, Wong G (2016). Genesis, evolution and prevalence of H5N6 avian influenza viruses in China. Cell Host Microbe.

[8] Yuan R, Wang Z, Kang Y, Wu J, Zou L, Liang L (2016). Continuing Reassortant of H5N6 subtype highly pathogenic avian influenza virus in Guangdong. Front Microbiol.

[9] Yang L, Zhu W, Li X, et al. Genesis and dissemination of highly pathogenic H5N6 avian influenza viruses[J]. J Virol. 2017;91(5):e02199–16. 10.1128/JVI.02199-16 PMC5309950 28003485

[10] Briand FX, Schmitz A, Ogor K, et al. Emerging highly pathogenic H5 avian influenza viruses in France during winter 2015/16: phylogenetic analyses and markers for zoonotic potential. Euro Surveill. 2017;22(9):1–11[J]. 10.2807/1560-7917.ES.2017.22.9.30473 PMC5356430 28277218

[11] Heine HG, Foord AJ, Wang J, Valdeter S, Walker S, Morrissy C (2015). Detection of highly pathogenic zoonotic influenza virus H5N6 by reverse-transcriptase quantitative polymerase chain reaction. Virol J.

[12] Qi X, Cui L, Yu H, et al. Whole-genome sequence of a Reassortant H5N6 avian influenza virus isolated from a live poultry market in China, 2013[J]. Genome Announc. 2014;2(5):e00706–14. 10.1128/genomeA.00706-14 PMC4161740 25212611

[13] Shen H, Wu B, Chen Y, Bi Y, Xie Q (2015). Influenza a(H5N6) virus Reassortant, southern China, 2014. Emerg Infect Dis.

[14] Wong FY, Phommachanh P, Kalpravidh W, Chanthavisouk C, Gilbert J, Bingham J (2015). Reassortant highly pathogenic influenza a(H5N6) virus in Laos. Emerg Infect Dis.

[15] Jiang H, Wu P, Uyeki TM, He J, Deng Z, Xu W (2017). Preliminary epidemiologic assessment of human infections with highly pathogenic avian influenza a(H5N6) virus, China. Clin Infect Dis.

[16] Kim HK, Jeong DG, Yoon SW (2017). Recent outbreaks of highly pathogenic avian influenza viruses in South Korea. Clin Exp Vaccine Res.

[17] Beerens N, Koch G, Heutink R, et al. Novel highly pathogenic avian influenza A (H5N6) virus in the Netherlands, December 2017[J]. Emerg Infect Dis. 2018;24(4):770. 10.3201/eid2404.172124 PMC5875252 29381134

[18] Noh JY, Lee DH, Yuk SS, Kwon JH, Tseren-Ochir EO, Hong WT (2018). Limited pathogenicity and transmissibility of Korean highly pathogenic avian influenza H5N6 clade 2.3.4.4 in ferrets. Transbound Emerg Dis.

[19] Miyoshi M, Komagome R, Yamaguchi H, Ishida S, Nagano H, Ohnishi A (2018). Administrative laboratory findings for highly pathogenic avian influenza virus a (H5N6) in individuals engaged in a mass culling of poultry, Hokkaido, Japan, 2016. Jpn J Infect Dis.

[20] Si YJ, Lee IW, Kim EH, et al. Genetic characterisation of novel, highly pathogenic avian influenza (HPAI) H5N6 viruses isolated in birds, South Korea, November 2016[J]. Euro Surveill. 2017;22(1). 10.2807/1560-7917.ES.2017.22.1.30434 PMC5388099 28079520

[21] Lee EK, Song BM, Lee YN, Heo GB, Bae YC, Joh SJ (2017). Multiple novel H5N6 highly pathogenic avian influenza viruses, South Korea, 2016. Infect Genet Evol.

[22] Naguib MM, Graaf A, Fortin A, et al. Novel real-time PCR-based patho-and phylotyping of potentially zoonotic avian influenza A subtype H5 viruses at risk of incursion into Europe in 2017[J]. Euro Surveill. 2017;22(1). 10.2807/1560-7917.ES.2017.22.1.30435 PMC5388100 28084214

[23] Butler J, Stewart CR, Layton DS, Phommachanh P, Harper J, Payne J (2016). Novel Reassortant H5N6 influenza a virus from the Lao People's Democratic Republic is highly pathogenic in chickens. PLoS One.

[24] Kwon JH, Lee DH, Swayne DE, Noh JY, Yuk SS, Erdene-Ochir TO (2017). Reassortant clade 2.3.4.4 avian influenza a(H5N6) virus in a wild mandarin duck, South Korea, 2016. Emerg Infect Dis.

[25] Hui Kenrie P. Y., Chan Louisa L. Y., Kuok Denise I. T., Mok Chris K. P., Yang Zi-feng, Li Run-feng, Luk Geraldine S. M., Lee Elaine F., Lai Jimmy C. C., Yen Hui-ling, Zhu Huachen, Guan Yi, Nicholls John M., Peiris J. S. Malik, Chan Michael C. W. (2017). Tropism and innate host responses of influenza A/H5N6 virus: an analysis of ex vivo and in vitro cultures of the human respiratory tract. European Respiratory Journal.

[26] Thanh HD, Tran VT, Nguyen DT, Hung VK, Kim W (2018). Novel reassortant H5N6 highly pathogenic influenza a viruses in Vietnamese quail outbreaks. Comp Immunol Microbiol Infect Dis.

[27] Monne I, Ormelli S, Salviato A, De Battisti C, Bettini F, Salomoni A (2008). Development and validation of a one-step real-time PCR assay for simultaneous detection of subtype H5, H7, and H9 avian influenza viruses. J Clin Microbiol.

